# A practice-based trial of blood pressure control in African Americans (TLC-Clinic): study protocol for a randomized controlled trial

**DOI:** 10.1186/1745-6215-12-265

**Published:** 2011-12-22

**Authors:** Antoinette Schoenthaler, Leanne Luerassi, Jeanne A Teresi, Stephanie Silver, Jian Kong, Taiye Odedosu, Samantha Trilling, Anna Errico, Oshevire Uvwo, Kimberly Sebek, Adetutu Adekoya, Gbenga Ogedegbe

**Affiliations:** 1 Center for Healthful Behavior Change, New York University School of Medicine, Department of Medicine, Division of General Internal Medicine, New York, NY; 2 Research Division, Hebrew Home at Riverdale, New York, NY; 3 Columbia University Stroud Center, Faculty of Medicine and New York State Psychiatric Institute, New York, NY; 4 Bellevue Adult Primacy Care Practice, Bellevue Hospital Center, New York, NY

**Keywords:** Hypertension, African American, Therapeutic Lifestyle Changes, Practice-based trial

## Abstract

**Background:**

Poorly controlled hypertension (HTN) remains one of the most significant public health problems in the United States, in terms of morbidity, mortality, and economic burden. Despite compelling evidence supporting the beneficial effects of therapeutic lifestyle changes (TLC) for blood pressure (BP) reduction, the effectiveness of these approaches in primary care practices remains untested, especially among African Americans, who share a disproportionately greater burden of HTN-related outcomes.

**Methods/Design:**

This randomized controlled trial tests the effectiveness of a practice-based comprehensive therapeutic lifestyle intervention, delivered through group-based counseling and motivational interviewing (MINT-TLC) versus Usual Care (UC) in 200 low-income, African Americans with uncontrolled hypertension. MINT-TLC is designed to help patients make appropriate lifestyle changes and develop skills to maintain these changes long-term. Patients in the MINT-TLC group attend 10 weekly group classes focused on healthy lifestyle changes (intensive phase); followed by 3 monthly individual motivational interviewing (MINT) sessions (maintenance phase). The intervention is delivered by trained research personnel with appropriate treatment fidelity procedures. Patients in the UC condition receive a single individual counseling session on healthy lifestyle changes and print versions of the intervention materials. The primary outcome is within-patient change in both systolic and diastolic BP from baseline to 6 months. In addition to BP control at 6 months, other secondary outcomes include changes in the following lifestyle behaviors from baseline to 6 months: a) physical activity, b) weight loss, c) number of daily servings of fruits and vegetables and d) 24-hour urinary sodium excretion.

**Discussion:**

This vanguard trial will provide information on how to refine MINT-TLC and integrate it into a standard treatment protocol for hypertensive African Americans as a result of the data obtained; thus maximizing the likelihood of its translation into clinical practice.

**Trial Registration:**

Clinicaltrials.gov NCT01070056

## Background

The disproportionately high prevalence of hypertension (HTN) and cardiovascular disease mortality in African Americans is well documented [1]. The age-adjusted prevalence estimate of HTN in African Americans is 31.8%, compared to 23.3% for whites [2], making African Americans the most highly afflicted group in the United States [3]. Hypertension is the single most common explanation for mortality disparities between African Americans and whites [4]. Fortunately, there is evidence that increased blood pressure (BP) control produces cardiovascular benefits in both African Americans and other ethnic groups [5].

Barriers that hinder adequate BP control exist at the individual level, especially in minority populations [6-8]. Potentially reversible individual-level factors include adverse lifestyle behaviors such as lack of physical activity, high sodium intake, poor intake of fruits and vegetables, and obesity. Several studies have confirmed the negative association between adverse lifestyle behaviors and blood pressure [9-14]. The *efficacy *of lifestyle interventions in the prevention and treatment of HTN is well proven [12-20]. Well-studied lifestyle approaches include weight loss;[21-23] restriction of sodium intake;[9,15,17,20,24,25] a diet rich in low-fat dairy and, fruits and vegetables;[18,25,26] limited alcohol intake;[27,28] and regular exercise [23,29-31]. For example, SBP/DBP reductions of 5.5/3.0 mm hg (comparable to those observed in drug trials) were achieved in the Dietary Approaches to Stop Hypertension (DASH) study, where patients were randomized to receive a diet rich in fruits and vegetables and higher in low-fat dairy products versus a standard diet [18]. The BP reduction was particularly high in African Americans in the DASH trial. A later lifestyle trial, PREMIER, showed that lifestyle changes were more effective in BP reduction than advice only [16]. Further analysis of PREMIER data indicated that BP reductions were similar or higher among African-American patients compared to whites;[32] and these intervention effects were maintained 18 months post-intervention [19]. Other trials such as the Trials of Mild Hypertension Study, Hypertension Control Program, Trial Of Nonpharmacologic interventions in the Elderly (TONE), Trials of Hypertension Prevention-Phase II, Diet, Exercise and Weight Loss Intervention Trial and the Primary Prevention of Hypertension have all reported similar beneficial effects of lifestyle changes on BP reduction [12,13,15,17,19,33-36]. Based on this evidence, non-pharmacologic approaches to BP control have been adopted as standard treatment in hypertensive patients by the Joint National Committee on Detection, Evaluation and Treatment of Hypertension (JNC)-5, JNC-6, and JNC-7 treatment guidelines [5,37,38]. However, all of these studies were conducted in clinical settings, under controlled conditions. If these interventions cannot find a place in clinical practice settings, their impact on public health will be negligible. This is particularly true for African Americans, given their poorer BP control compared to other racial/ethnic groups,[39] as well as their low participation rates in these trials. The current study represents an advance in the non-pharmacologic treatment of HTN in high risk populations because it emphasizes the translation of an empirically-validated TLC intervention into clinical practice.

### Study Aims

The primary aim of the Therapeutic Lifestyle Changes in Clinic (TLC-Clinic) trial is to test the effectiveness of a practice-based comprehensive therapeutic lifestyle intervention, delivered through group-based counseling and motivational interviewing (MINT-TLC) versus a Usual Care control condition (UC) in reducing BP, among 200 low-income, poorly controlled hypertensive African-American patients. The secondary aims are to evaluate the effect of MINT-TLC versus UC, on changes in the following lifestyle behaviors from baseline to 6 months: a) physical activity, b) weight loss, c) number of daily servings of fruits and vegetables and d) 24-hour urinary sodium excretion. In addition, a secondary aim will evaluate the effect of MINT-TLC versus UC on BP control at 6 months.

We hypothesize that patients randomized to the intervention condition (MINT-TLC) compared to those randomized to the UC condition, will exhibit a greater reduction in systolic BP (SBP) and diastolic BP (DBP) at 6 months; greater levels of physical activity; greater percent change in weight; increased intake of fruits and vegetables; reduced 24-hour urinary sodium excretion; and a higher proportion of patients will have adequate BP control at 6 months. This study is one of few trials to test the effectiveness of this approach among minority populations who receive care in primary care practices.

## Methods/Design

### Study Design

TLC-Clinic is a randomized controlled trial (RCT) with two conditions: Intervention condition (MINT-TLC) and Usual Care condition (UC). Approximately 200 African-American patients with uncontrolled HTN will be randomly assigned to either the MINT-TLC or UC conditions. The MINT-TLC intervention is based on established clinical practice guidelines for prevention and treatment of HTN, which recommends weight loss (if overweight), limiting sodium and alcohol intake, regular physical activity, and eating a low-fat diet that is rich in fruit and vegetables. MINT-TLC will be conducted by trained Health Educators. Patients attend 10 weekly group classes (intensive phase) followed by individual monthly motivational interviewing (MINT) sessions for 3 months (maintenance phase). Makeup sessions are built in after sessions 4 and 9 for group members who missed any of the previous sessions. Assessments are conducted at baseline (visit 1), 3 months (visit 2), and 6 months (visit 3). Participants receive $20 at the completion of baseline assessment, $10 at the 3-month visit, and $20 at the completion 6-month visit ($50 total) for their time and effort in the study. All participants also receive free metro cards to help facilitate transportation to and from the study site for the individual counseling visit (UC condition) or group classes (MINT-TLC). The primary and selected secondary outcomes are assessed at the 3 month visit, and all outcomes are assessed at the 6-month follow-up visit.

### Study Sites and Population

This study is being conducted within the Ambulatory Care Practice of the Bellevue Hospital Center (BHC), the largest comprehensive diagnostic center within the New York City Health and Hospitals Corporation. Approximately 20,000 patients receive treatment annually at BHC's adult medicine primary care practice, with just under 60,000 visits in 2009. To be eligible, patients must self-identify as black/African American, be at least 18 years old, currently receive care within the Ambulatory Care Practice and have had at least two appointments in the past year, have a diagnosis of HTN with uncontrolled BP on at least two visits in the past year (SBP ≥ 140 mmHg *or *DBP ≥ 90 mmHg or SBP ≥ 130 mmHg *or *DBP ≥ 80 mmHg for patients with diabetes or chronic kidney disease). In addition, all patients must have uncontrolled BP at the time of the consent visit, as measured by WatchBP (Microlife; Golden, CO), an oscilliometric validated BP monitor [40,41]. Patients are excluded if they are non-English speaking, participate in other HTN trials, have an arm circumference > 42 cm, have cognitive impairment based on the Comprehensive Assessment and Referral Evaluation Dementia Diagnostic Scale (CARE-DIAG) score ≥ 6 for patients age 60 and older,[42,43] are unwilling/unable to comply with the study protocol (either self-selected or by indicating that they could not complete all requested tasks including attending intervention classes if they were to be randomized to the MINT-TLC group), or unwilling/unable to provide informed consent. All patients who agree to take part in the study are asked to provide informed consent. The study was approved by the Institutional Review Boards of New York University and Bellevue Hospital Center. The study is monitored by a Data Safety Monitoring Board.

#### Recruitment

Three main approaches are used to recruit patients into this study. First, potentially eligible patients are identified through a review of electronic medical records (EMR) using the ICD-9 billing codes for HTN (401-401.9). The physicians of eligible patients are notified of their patients' potential eligibility, and then asked permission to enroll their patients in this study. After consent is obtained from the physicians, eligible patients are approached by research assistant's (RA) at their next clinic appointments for screening, consent and baseline visit. Second, physicians are asked to identify hypertensive patients in their practices through the use of ICD-9 billing codes. Once a patient is identified who fulfills the inclusion criteria, the RA calls the patient and invites him or her to complete the eligibility screen, and if eligible, to consent and complete the baseline visit. Study investigators (GO, JR, TO) also conduct in-service visits at the study sites prior to patient enrollment and every 3 months thereafter to explain the study rationale, its significance and procedures. At this time, physicians are given a business card that lists eligibility criteria and contact information for the RAs. Finally, flyers are hung in the waiting room of the Ambulatory Care Practice and RAs approach patients about the study in the waiting areas.

#### Randomization

After completion of consent procedures and baseline data collection, a list of patient identification numbers is sent on a weekly basis to the data management team to be randomized in a 1:1 ratio to either the MINT-TLC or UC group. This project has a hierarchical study design with each patient nested within a primary care physician and patients are recruited at different times. Randomization is within primary care physician. Therefore, a randomization algorithm developed by the Data Coordinating Center at the Research Division of the Hebrew Home at Riverdale was used; this algorithm has yielded balanced groups for numerous studies. This randomization procedure is carried out using a SAS macro after a subject completes the baseline interview. A random number from 0 to 1 is used to determine the assignment group. The standard cut score is set at 0.5 for the first n subjects from the same provider, 4 for this study. Those who receive a random number between 0 and 0.5 are assigned to the control group and those with a random number greater than 0.5 are assigned to the intervention group. The balance between the groups within each provider is weighted carefully after the total number of subjects from a group reaches a pre-specified number. If more than the n subjects are randomized initially, the cut score for the next subject is equal to the ratio of the experimental group (n1) to the subjects already randomized (m) for that group (n1/m). The cut score is adjusted according to the previous number of subjects randomized.

Patients are informed of the results of randomization by an RA in person or by phone; at this time, the RA discusses the intervention/counseling session schedule, and answers any additional questions. Given the nature of the intervention, it is impossible to blind the RAs to the assigned condition. However, the dimension that the RA's knowledge of the assigned condition could possibly affect is the BP measurement - the primary outcome of this study. To reduce the potential for this bias, we use an automated BP device (WatchBP). Neither the RA nor the patients see the measurements during this process, which prevents the RA from influencing the BP readings.

### Description of the Intervention

#### MINT-TLC Condition

This intervention is based on established clinical practice guidelines for prevention and treatment of HTN, which recommends weight loss (if overweight), regular physical activity, limiting and/or reducing sodium and alcohol intake, and eating a low-fat diet that is rich in fruit and vegetables [5]. Patients attend 10 weekly group classes over 12 weeks (intensive phase) followed by monthly, individual telephone-based MINT sessions for 3 months (maintenance phase). Makeup sessions are built in after session 4 and session 9 for group members who missed any of the previous sessions. The methodology, structure and the content of the group classes is patterned after PREMIER and Healthy Eating and Lifestyle Program (HELP) developed by Kumanyika et al. [44] The HELP program is based on the group counseling approach used in the Trial Of Nonpharmacologic interventions in the Elderly (TONE) trial, and was culturally tailored for African Americans. PREMIER is a comprehensive multi-component lifestyle program which incorporates the DASH dietary pattern [18]. The addition of three individual MINT sessions to this protocol was carefully chosen to mimic the structure of the two major trials that have tested the efficacy of comprehensive TLC in hypertensive patients [18,20]. The methodology, structure and content of the individual MINT sessions are patterned after our recently completed study in the same population [45].

##### Intensive Phase (Group Classes)

The group classes are conducted by Health Educators trained in behavioral counseling approaches. Each class lasts between 60 and 90 minutes, and is structured so that homework from previous sessions, a review of the goals participants set the previous week, feedback and trouble shooting for skill-building and new content is covered in each session. The sessions focus on developing skills, goal-setting and generating strategies for behavior change as well as support for relapse prevention. Each session follows a similar structure and includes the following components: 1) Overview of HTN and antihypertensive medications; 2) DASH eating plan 3) Goal setting and healthy living diaries; 4) Serving sizes, portion control and food labels (with emphasis on sodium monitoring); 5) Physical activity; 6) Building skills for meal planning and shopping; 7) Recipe modification and eating away from the home; 8) Stress Management; 9) Eating triggers and mindful eating; and 10) Planning for lasting change. The group classes are structured to be very interactive with patient input and smaller group activities that foster problem solving, support, and program ownership. The specific content of classes is shown in Table 1.

**Table 1:** Major Content of TLC Intervention Sessions

Session	Content
**1. Getting Started & Blood Pressure Overview**	Introduction to the program. Education about blood pressure and hypertension. Discussion of risk factors for hypertension and behavioral approached to controlling hypertension.

**2. Medications**	Introduction to medications for HTN. Myths and Facts about HTN medications, tips for taking charge of medications, and understanding medication labels. Discussion of current medication taking behavior and goals for adherence. Introduction to the Medication Diary. At-Home Activity: Medication Diary.

**3. DASH Eating Plan & Introduction to Goal Setting**	Introduction to the DASH Eating plan. Discussion of DASH pyramid, food groups and servings. SMART goal setting for healthy eating goal. Introduction to Healthy Living Diary (food and activity diary). At-Home Activity: Healthy living diary, Medication diary.

**4. Food Labels & Sodium**	Discussion about reading food labels and nutrition facts. Facts about salt and sodium, and tips for how to lead a low sodium life. Revisiting healthy eating goal and introduction of rewards. At-Home Activity: Healthy living diary, Medication diary.

**5. Physical Activity & Stress Management**	Introduction to physical activity. Discussion of FITT guidelines, impact of exercise on HTN, finding time for physical activity. Physical activity goal setting. Discussion of stress and health. Quiet time activity. At-Home Activity: Healthy living diary, Medication diary.

**6. Serving Sizes and Portion Control**	Review of medications and DASH eating plan. Discussion about serving sizes, portion control and the plate method. Revisiting the physical activity goal. At-Home Activity: Healthy living diary, Medication diary.

**7. Meal Planning & Shopping**	Discussion of the buddy system and physical activity. Discussion of planning for meals and tips for shopping. Checking in on goal progress. At-Home Activity: Healthy living diary, Medication diary.

**8. Eating Triggers & Eating Awareness**	Discussion of eating triggers and eating awareness. Mindful eating activity. Revisit health eating goal. At-Home Activity: Healthy living diary, Medication diary.

**9. Specials Occasions, Eating Out &Recipe Substitutions**	Discussion of strategies for attending special occasions and eating out. Recipe substitutions. Revisit physical activity goal. At-Home Activity: Healthy living diary, Medication diary.

**10. Planning for Lasting Change**	Review of overall program content. Discussion of what worked with medication adherence. Adopting the attitude "It's not a diet it's a lifestyle." Discussion of physical activity throughout the year. Planning for lasting change activity.

##### Extended Phase (Monthly Individual MINT Sessions)

The individual telephone-based MINT sessions are conducted by the trained Health Educators. The goal of this phase is to help patients focus on problem solving, goal setting, and prevention of relapse with regard to each of the therapeutic lifestyle changes adopted during the intensive phase. The sessions focus on individual needs to tailor intervention strategies to the patient's personal context including social support, specific behavior change goals, problem-solving, and maintaining motivation during challenging situations. Each session assumes the following steps: 1) assessing the patient's motivation and confidence in engaging in a given behavior - physical activity, dietary restriction of sodium, increased intake of fruits and vegetables, stress management or adherence to prescribed antihypertensive medications; 2) eliciting barriers and concerns about adoption of each lifestyle modification; 3) summarizing in a non-threatening manner the 'pros' and 'cons' of the patient's concerns, thereby eliciting positive self-motivational statements about the behavior; 4) providing a menu of options to the patient based on the nature of barriers elicited from the patient; 5) assessing each patient's values and goals, in order to help the patient link current health behavior patterns to core values and life goals. The session ends with a global summary of what was discussed and a clarification of an action plan agreed upon. Each session lasts up to 30 minutes and is conducted with the aid of a standardized counseling script [45].

##### Monitoring treatment fidelity (intensity and the dose of the intervention)

Adherence to the intervention protocol is an important aspect of this study given its intensity. At the trial's inception, the Health Educators participated in a 3-day training that consisted of an overview of the behavioral framework of the intervention; the curriculum; and basic group facilitation skills taught through role-playing and didactic instruction. In addition, the Health Educators participated in a 2-day training on motivational interviewing skills including an overview of the basic principles of MINT techniques, development of problem-solving approaches, and practice sessions in the form of role-plays. Weekly meetings with study staff are held to discuss the sessions and plan for the subsequent week. Booster training sessions are also conducted monthly to minimize drift in MINT counseling skills.

All group and individual MINT sessions are audio-taped and used for two related purposes: (1) to provide feedback and ongoing training/supervision of the Health Educators and (2) systematic review for treatment fidelity. The relatively immediate review and use of the recordings serve to facilitate the feedback and coaching process to develop and maintain the skills of the individual Health Educators, a training approach that has proven to be most effective in previous studies of MINT [46]. Audio files are also archived to allow for systematic sampling of 30% of the MINT sessions, which will be coded in order to assess adherence to MINT principles using the Motivational Interviewing Treatment Integrity (MITI) scale [47]. The MITI scoring system was recently validated in an addiction treatment setting and found to have satisfactory inter-rater reliability and good convergent and discriminative validity [47]. In addition, this session archive will be used for future process evaluation to identify the important components of MINT used in the health behavior setting, as suggested by other researchers [48]. Finally, we record completion of the food diary and homework assignments as well as attendance of group classes and note the compliance and attrition rates.

### Description of the Control Condition/Usual Care

Patients randomized to the UC condition receive standard HTN treatment recommendations as determined by their physicians. In addition, they receive a 30-minute individual counseling session on therapeutic lifestyle modification, similar to PREMIER [16]. To match the MINT-TLC group for content of intervention material, those in the UC group also receive print versions of the intervention curriculum that is distributed in the group classes.

### Outcomes assessments

The primary outcome is change in SBP and DBP at 6 months. Secondary outcomes include: intake of fruits and vegetables; level of physical activity; percent change in weight; level of 24-hour urine sodium excretion; and proportion of patients with adequate BP control at 6 months. Study assessments for primary outcomes are completed at the baseline visit, 3 and 6 months; data collection intervals are given in Table 2. All assessments are performed by trained RAs and divided into three categories: (1) physiological and anthropometric measures, (2) self-report measures and (3) chart data. Table 2 summarizes the measures according to their timeline.

**Table 2:** Study measures by modality

MEASURES	Baseline	3 Months	6 Months
**PHYSIOLOGICAL**			

Office BP Measurements	X	X	X

24-hour ABPM	X		X

Height, Weight, and Body Mass Index	X		X

Blood and Urine Specimen	X		X

**SELF-REPORT**			

Patient Demographics	X		X

Cognitive Screen for participants ≥ 60 yrs (CARE-DIAG)	X		

Diet (Food Frequency questionnaire)	X	X	X

Physical Activity (IPAQ Long Version)	X	X	X

**CHART DATA**			

Frequency of Clinic Visits and Medication Changes	X		X

Hypertension characteristics	X		X

Medical Comorbidity (Charlson Comorbidity Index)	X		X

### Primary Outcome

#### Blood Pressure

At baseline, 3 blood pressure (BP) readings are taken by a trained RA using an automated BP monitor (WatchBP Office Device; Microlife; Golden, CO) with the patient seated comfortably for 5 minutes before measurement, following AHA guidelines [49]. The average of the 3 readings is used as the measurement for each visit. Uncontrolled BP is defined as an average SBP ≥ 140 mm Hg or DBP ≥ 90 mm Hg. The criteria for those with co-morbid diabetes or kidney disease are SBP ≥ 130 mm Hg or DBP ≥ 80 mm Hg.

### Secondary Outcomes

*Dietary intake of fruits and vegetables *is assessed via a web-based graphical food frequency questionnaire (FFQ), which was adapted from the FFQ developed by Fred Hutchinson Cancer Research Center [50]. The instrument assesses how often, and in what amount patients eat a list of over 125 food items. The Graphical FFQ assesses portion size using photographs in order to get accurate responses.

*Physical activity *is assessed via a computerized version of the International Physical Activity Questionnaire (Long IPAQ) [51]. The Long IPAQ is a 32-item measure that allows for evaluating the amount of health-related vigorous and moderate-intensity physical activity as well as sedentary behaviors and physical activity limitations in adults over seven days. The number of hours and minutes per day participants report spending in various types of physical activity is multiplied by the average metabolic equivalent (METs) of each category and summed to calculate energy expenditure as kcal/kg/minutes/week.

*Height and weight *is measured without shoes and with lightweight clothes using a stadiometer and a validated digital scale (WB-800S Digital Medical Weight Scale; Tanita Corporation of America, Inc.; Arlington Heights, Illinois), respectively. All measurements are recorded to the nearest 0.1 cm and 0.1 kg. These data are used to calculate and determine the patient's body mass index (BMI). The difference in weight from baseline to 6 months serves as a measure of weight loss. Height is only measured at the baseline visit while weight is measured at baseline and 6 months.

A *24-hour urine sample *is collected at baseline and 6 months for analysis of urinary sodium excretion. Trained RAs provide instructions and a collection container in the clinic. Patients are also provided with a collection log on which they record the start and stop time; any missed voids, spills, and any other problems with the collection. Patients with inadequate collections (e.g., < 20 hours or < 500cc or > 24 hours) are asked to repeat the process.

*BP control *is defined as an average BP (based on 3 readings with automated BP monitor) that fulfills the JNC-7 criteria of SBP < 130 and DBP < 80 mmHg (patients with diabetes or chronic kidney disease); or SBP < 140 and DBP < 90 mmHg (for all other patients).

### Chart Extraction Data

All patients have their medical records reviewed at baseline and at 6-months by study staff. Chart data extraction includes duration of HTN, evidence of target organ damage, changes in prescribed antihypertensive medications dosages (this reflects the intensity of treatment). Others include frequency of clinic visits and reasons for visits; and use of other medications that are known to affect BP such as NSAIDS and hormone replacement therapy. Patients in this population have comorbid illnesses, which may affect BP levels - the primary outcome of this study. As such, we will use the Charlson Comorbidity Index (CCI)[52] to adjust for both the confounding effects of comorbidity on BP and their non-uniform prognostic impact on patients. The CCI is a weighted index for prospectively classifying comorbid conditions, which takes into account the number and seriousness of comorbid diseases.

Data on *patient socio-demographic characteristics *are also collected for description of the cohort and to examine effects of these factors on BP control. Variables include age, gender, education level, income, marital status, employment status, and health insurance status.

### Analysis

#### Sample Size and Power Analysis

The sample size is based on the number of patients required to provide adequate power to test the primary hypothesis of group differences in SBP and DBP. The power calculations assumed separate analyses of SBP and DBP; however, the use of multivariate analyses of variance (MANOVA) to examine the primary outcomes simultaneously, assuming that they are correlated was also considered. Power is expected to be greater with the use of MANOVA. However, we will use Full Information Maximum Likelihood estimation procedures for the primary analyses, thereby allowing us to include participants who do not complete the follow-up assessment in the analysis (on an intent-to-treat basis). The original power calculations were conservatively based on the number of participants who were anticipated to complete the follow-up assessment.

Based on our own clinical experience and estimates from other clinical trials in uncontrolled hypertensive patients,[16-18,20,53-57] we estimate that the cross-sectional standard deviations (SD) of SBP and DBP at baseline will be about 15/12 mm Hg. It was assumed that the SD at follow-up will be as much as 25% larger in the MINT-TLC group, and that the correlation between baseline and follow-up BPs will be 0.6 in the UC group and 0.4 in the MINT-TLC group (possibly as a result of heterogeneity of treatment response). Design effects of patients clustered within physicians has been found to be minimal in our experience; for example the intracluster correlation coefficient (ICC) was estimated to be 0.02 or less, and cluster sizes are relatively small. Clustering results in reduction in power and increased requirements for sample size. The variance inflation factor (VIF) used in power calculations involving clustering depends on the size of the cluster (number of patients treated by each physician) and ICC. Power calculations were examined in sensitivity analyses (not shown), assuming that heterogeneous variances may require modeling. The original power calculations were updated using actual data from the beginning of the trial. It was found that the average cluster size was 1.55 (SD = 1.39, ranging from 1 to 10 clusters and the total number of physicians was 92). The following assumptions were used in the power calculations: σ (population standard deviation) = 15 for SBP and 12 for DBP; α = 0.05, two-tailed; R = 0.9 (reliability); g = 2 (groups); n = 3 (waves, t = 0, 3 and 6 month); $s_{x}^{2}$(variance of time) = 0.125; inflation factor (IF) = 1+(clustersize-1)*ICC with clustersize = 1.55 and ICC = 0.01.

Thus, 100 subjects per group will provide 80% power to detect a 6 to 7 mm Hg differential change in SBP and a 5 to 6 mm Hg differential change in DBP, based on testing the Time (pre through post-intervention waves) × Group interaction in a mixed model, allowing for heterogeneous variances, serial correlations and modeling of design effects. As described below, analyses will be performed under an intent-to-treat design; therefore all patients, including those who drop out of the study will be invited back for the final assessments.

### Analysis of the Primary Aim

The primary pre-planned analyses are to examine SBP and DBP separately; however, if the two variables are correlated, MANOVA will be used to test the hypothesis that those assigned to MINT-TLC will, on average, exhibit greater 6-month decreases in SBP and DBP than those assigned to the UC condition. A mixed (fixed and random) effects model using SAS Proc Mixed is the method planned for use. Such models permit modeling heterogeneous group variances and serial correlations over time as well as the design effects of clustering. Moreover, the use of the missing data algorithms within the mixed procedure permits the inclusion of all individuals with at least one wave of data. The primary test concerns the Group × Time interaction, and the resulting F-test will provide the primary "intent-to-treat" test of the hypothesis. If this is statistically significant at the two-tailed α = 0.05 level, the magnitude of the treatment effect will be estimated with 95% Confidence Interval (CI), separately for SBP and DBP. The expectation is that the randomization of patients to treatment arm and the absence of significant selection and/or attrition biases will obviate the need for any covariates in the analysis. This has been the normative experience of the data coordinating center in over 100 randomized trials. However, in the unlikely event that imbalances are identified, propensity score analyses or other methods will be used to condition on the covariates. The predicted values of those analyses will be included as covariates together with the group by covariate interactions in the model. This procedure is most often applied in quasi-experimental and observational studies.

## Discussion

### Study Implementation: Challenges and Lessons Learned

In the first few months of implementing the study, we encountered several "real world barriers" that have made us reevaluate the suitability of the TLC-Clinic protocol as originally conceived. Several of the challenges faced by the TLC-Clinic trial relate to the difficulties involved in translating an empirically-validated therapeutic lifestyle intervention into clinical practice. Moving away from a tightly controlled academic research center to a practice-based setting adds a new level of complexity as researchers must contend with the need to uphold the methodological rigor of a randomized clinical trial while maintaining the flexibility to work within a busy primary care practice.

To maintain this balance, the investigative team holds biweekly strategic meetings where barriers and solutions are discussed within the context of the study's aims. Moreover, we adopted several successful strategies based upon our experiences with implementation of similar procedures from previous clinical trials in practice-based settings [55,56]. As a result, we have had little difficulty in delivering the intervention at the Ambulatory Care Practice and this success can be attributed to the following factors; first, the presentation of the study to the clinic director, primary care providers and allied health workers in the practice on multiple occasions during staff meetings in order to gain the buy-in from providers and clinic staff. Second, we had one of the primary care providers to serve as co-investigator on the study as a physician champion for the study within the practice. Third, we engaged the medical assistants in recruitment of patients and we arranged for a dedicated space to deliver the interventions to the patients by asking the director to provide available space within the practice.

The two main barriers to date are patient recruitment and the poor attendance rates of the group sessions and the low participation rates for the telephone-based MI counseling sessions. Below, we summarize the strategies we have employed to address these challenges as resource for other researchers who are interested in translating efficacious lifestyle interventions into community-based practices.

### Recruitment Challenges

One of the most frequently encountered barriers to recruitment in a primary care clinic is the limited availability of private space to conduct patient screenings, consent, and assessments. Due to a high patient caseload, exam rooms are often in use, making it difficult for RAs to secure a private space to meet with study participants. As a result, RAs are often faced with the task of juggling space and time constraints so as not to interfere with the clinic workflow or compromise patient rapport. The RAs have been flexible and quick to adapt to such situations to ensure that patients are comfortable and that the interviews remain confidential while not interfering with their medical visits. We have also developed a rapport with clinic staff that assist in identifying empty exam rooms, when available.

A second recruitment challenge that is more likely to be encountered when recruiting from a community clinic is related to the inclusion criteria chosen to select the study population. It quickly became apparent that the original criterion that was contingent on patients receiving care within the NYU Ambulatory Care Network for *at least *one year restricted the pool of eligible patients substantially, resulting in recruitment numbers below the target goal. In response, the inclusion criterion was changed to "currently receiving primary care within the NYU Ambulatory Care Network and has had at least two primary care appointments in the past year" and "at least one high BP reading in the past year" which resulted in a significant increase in patients meeting the initial eligibility criteria. Several other changes have been made to the eligibility criteria to boost recruitment including reevaluating patients with BP that was too high at the initial screening and including patients with a history of substance abuse if the patients' provider agrees they would be a good candidate.

The methods of identifying eligible patients for the study also presented several challenges. Based on previous experience,[55] we planned to capitalize on the clinic's EMR system to prescreen potentially eligible participants based on the study inclusion criteria. During the initial months of the trial implementation, a list of eligible patients was created each week indicating patient's appointment time and physician name. Despite having this information, the number of potential participants recruited from this list was extremely low and is no longer used as a recruitment strategy for three key reasons. First, the EMR system is not equipped to conduct a refined search strategy and so, a significant amount of staff time was needed to cross check the information on the list with individual medical records to determine initial eligibility. Second, a high percentage of patients on the refined list broke their scheduled appointment. To address the latter issue, we created an 'opt-out letter' whereby patients who did not show up for their appointment were mailed a letter, signed by his/her physician, two weeks before the patient's next appointment. However, this too produced a low yield and is no longer used as a recruitment strategy. Third, physicians often see patients back-to-back throughout the day (average of 9 patients per session, of which 1 is a new patient) and do not have the time to speak with the RAs about potentially eligible study participants. In addition, due to the inherent nature of the time-constrained visit as well as competing medical priorities, physicians are often unable to directly encourage patients to enroll in the study. However, without the support from their primary care physicians, some patients are less inclined to learn more about the study.

To date, "cold recruiting" patients in the waiting room has proven to be the most effective recruitment strategy. However, while numerous patients in the waiting area express initial interest in the TLC program, many do not meet the initial screening criteria due to active substance abuse, significant mental health disorders, not being a BHC primary care patient, missing BP data or no HTN diagnosis in the EMR, and limited English proficiency, with a majority of African patients speaking French Creole as their primary language. Many others cannot commit to attending classes at BHC on a weekly basis because they work full time or live in the outer boroughs and cannot afford to travel to Manhattan on a regular basis. For those patients who do meet initial criteria, the RA typically schedules a meeting at a later date to complete the consent and initial study visit. However, many patients are reluctant to come back to the clinic a second day and express disappointment that car fare is not provided for attending any of the study-related visits. Despite the many recruitment challenges we have encountered, approximately 60% of patients have been approached and cold-recruited in the clinic waiting rooms. The successful use of cold recruitment attests to the RAs ability to develop a trusting interpersonal relationship with patients, which is also reflected in the 83% retention rates for the 3 and 6-month follow-up visits.

### Mode of Intervention Delivery

The potential for low patient participation rates in the group classes and MINT counseling sessions is another real-world challenge encountered in this study. We have adopted several strategies to facilitate high participation rates, including offering classes in the clinic; determining a cohort's class schedule based on the most frequently selected time and date chosen by patients during the eligibility screen visit; and offering Saturday classes and make-up classes on an alternate day and time. Despite these efforts, approximately 50% of patients attend the weekly group classes, of which 35% have attended 9 to 10 of the classes (total of 10 classes). Most frequent reasons for low group class attendance include patients' reporting another time commitment or conflict, being too ill to travel to the classes on a weekly basis, inability to reach the patient to remind them of the class time and location, and patient's having difficulty arranging for paratransit services (e.g., Access-a-Ride) to bring them to the clinic on off hours (evenings and weekends).

Similarly, approximately 50% of patients have received the telephone-based MINT sessions to date. To increase the completion rate, Health Educators make four call attempts to each patient at different times of the day over a 2-week period before they are considered a missed appointment. However, it is often difficult to reach patients by phone as many participants have intermittent use of their phones and their numbers change frequently. Letters signed by the Health Educators are also sent to patients with the study coordinator's phone number. It is noteworthy that 47% of patients that attended at least 5 of the group sessions have completed at least 1 MINT session. Further, 42% of patients that attended no classes have completed at least 1 MINT session, which suggest that this intervention modality may be effective in certain subgroups.

Despite the many challenges we have experienced to date, implementation of this trial has presented us with the unique opportunity to question which intervention approaches work in real-world settings, where patients have competing priorities and the demands of life often outweigh the perceived benefits of participation. Based on our experiences implementing the TLC-Clinic trial, we would recommend future researchers conduct a formative evaluation prior to implementing the trial at the participating clinic sites.

A strength of formative evaluation is the involvement of the target population, which can create buy-in and limit potential barriers to acceptance among future participants. This is accomplished by soliciting input and acting on the suggestions from the pre-intervention implementation phase to the completion of the project. Formative evaluation can begin by surveying the physical space where participant recruitment and study visits would occur and documenting clinic workflow processes currently in place to determine how the study procedures may complement rather than disrupt this process. Semi-structured interviews and/or focus groups with patients and physicians can also be utilized to elicit physicians' attitudes about practice-based research, identify patient barriers and facilitators to attending intervention sessions, test the recruitment and data collection procedures, and identify any organizational practices or structural barriers that may inhibit implementation of the protocol as intended. A small, single arm study can also be conducted to pilot the individual intervention components and garner participant feedback on changes that could be made to make the approach more effective. Finally, throughout the trial, observational notes by study staff as well as short interviews with participants can be completed to provide ongoing evaluation of the acceptability of the intervention and fidelity to the protocol. This evaluation framework has shown to be successful in other large multi-component behavioral interventions [58,59].

In response to the implementation challenges we have encountered to date, we recently incorporated patient semi-structured interviews into the end of the group classes to garner feedback about the structure and content of the classes, and suggestions for improvement. Data collected from the exit interviews as well as the research staff's experiences will allow us to look inside the 'black box' of the intervention design and delivery to identify those components that are considered by patients to be the most beneficial so that future lifestyle interventions in primary care practices can be tailored to the needs of the population.

### Trial Status

The TLC-Clinic trial began in February 2010 in the Bellevue Ambulatory Care Practice within Bellevue Hospital Center (BHC). To date, we have recruited a total of 166 patients, of which 83 have been randomized to the MINT-TLC arm. Patient recruitment will be completed in approximately 2 months and patients will be followed for 6 months.

## List of abbreviations used

HTN: Hypertension; TLC: Therapeutic lifestyle changes BP: Blood pressure; MINT: Motivational Interviewing; UC: Usual care; RCT: Randomized control trial; DASH: Dietary Approaches to Stop Hypertension; TONE: Trial of Nonpharmacologic Interventions in the Elderly; HELP: Healthy Eating and Lifestyle Program; JNC: Joint National Committee; BHC: Bellevue Hospital Center; RA: Research assistant; EMR: Electronic medical record; SBP: Systolic blood pressure; DBP: Diastolic blood pressure; CARE-DIAG: Comprehensive Assessment and Referral Evaluation Dementia Diagnostic Scale; FFQ: Food Frequency Questionnaire; CCI: Charlson Comorbidity Index; VIF: Variance inflation factor; CI: Confidence Interval.

## Competing interests

The authors declare that they have no actual or potential competing interests.

## Authors' contributions

GO conceived of and designed the study and helped to draft the manuscript. AS drafted the manuscript and participated in the coordination of the study. LF participated in the design and coordination of the study and helped to draft the manuscript. JT and SAS participated in the design of the study and preliminary analysis, and helped to draft the manuscript. JK designed and programmed the randomization procedures, and performed randomization. JT and JK performed the power calculations. ST, AE, OU, KS, and AA participated in the implementation of the study and helped to draft the manuscript. All authors read and approved the final manuscript.
